# Ventriculoperitoneal Shunt Migration Inside the Gastric Lumen: A Rare Case Report

**DOI:** 10.7759/cureus.4453

**Published:** 2019-04-13

**Authors:** Jasdeep S Sidhu, Amrendra Mandal, Paritosh Kafle, Baikuntha Chaulagai, Vijay Gayam

**Affiliations:** 1 Internal Medicine, Interfaith Medical Center, Brooklyn, USA

**Keywords:** ventriculo-peritoneal shunt, percutaneous gastrostomy tube, migration

## Abstract

Ventriculoperitoneal (VP) shunt placement is one of the more common procedures in neurosurgery and has a variety of indications. However, shunt placement can be associated with multiple complications, one of which is proximal and distal shunt migration. There have been reported cases of migration of the distal end of a VP shunt from the intraperitoneal cavity into different organs resulting in a variety of complications. Most of the reported cases are the result of spontaneous migration. However, shunt catheter migration could be iatrogenic as well. We present a case of intragastric VP shunt migration in a patient following placement of a percutaneous endoscopic gastrostomy tube.

## Introduction

Ventriculoperitoneal (VP) shunt placement is a common procedure for the treatment of hydrocephalus. Bowel perforation is a rare complication associated with shunt catheter migration with an incidence of only 0.1% to 0.7% [1]. The colon is the most common site of bowel perforation [2-3]. Intragastric catheter migration is extremely rare with only a few reported cases [4-6]. Significant morbidity and mortality are associated with shunt migration; therefore, prompt diagnosis and treatment are critical.

## Case presentation

An 84-year-old male resident of a nursing home facility of Hispanic descent was brought to our emergency department (ED) for respiratory distress and altered mental status. He was intubated promptly on arrival to the ED. His past medical history was significant for intracranial aneurysm with bleeding following VP shunt placement, ischemic stroke with aphasia and paraplegia, and percutaneous endoscopic gastrostomy (PEG) tube placement. His vital signs and clinical laboratory results are presented in Table 1.

**Table 1:** Patient vital signs and clinical laboratory results

Vital Sign	Measurement	
Temperature	102.6 °F	
Heart rate	128 beats/minute	
Blood pressure	90/56 mmHg	
Respiratory rate	26 breaths/minute	
Oxygen saturation	88% (on Venturi mask with oxygen flow of 6 L/minute)	
Laboratory Analyte	Patient Value	Reference Range
WBC	12,400/µL	4,000–11,000/µL
PMN	88%	
Hb	7.1 g/dL	13.5–17.5 g/dL
MCV	98.5 µm^3^	80–100 µm^3^
Serum sodium	15.5 mEq/L	13.6–14.4 mEq/L
BUN	73 mg/dL	8–20 mg/dL
Lactic acid	2.3 mmol/L	0.5–1.9 mmol/L
AST	27.3 U/L	15–41 U/L
ALT	37.7 U/L	17–63 U/L
ALP	30.1 U/L	32–91 U/L

The clinical picture was suggestive of septic shock. We ordered a sepsis workup including two sets of blood cultures and urine culture. The patient was treated with aggressive intravenous fluid hydration and broad-spectrum antibiotics (vancomycin and meropenem).

**Figure 1:**
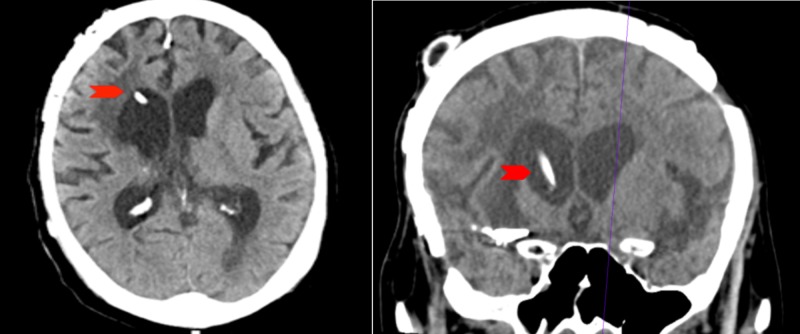
CT of the head showing VP shunt entering anterior horn of right lateral ventricle (red arrow) CT, computed tomography; VP, ventriculoperitoneal

**Figure 2:**
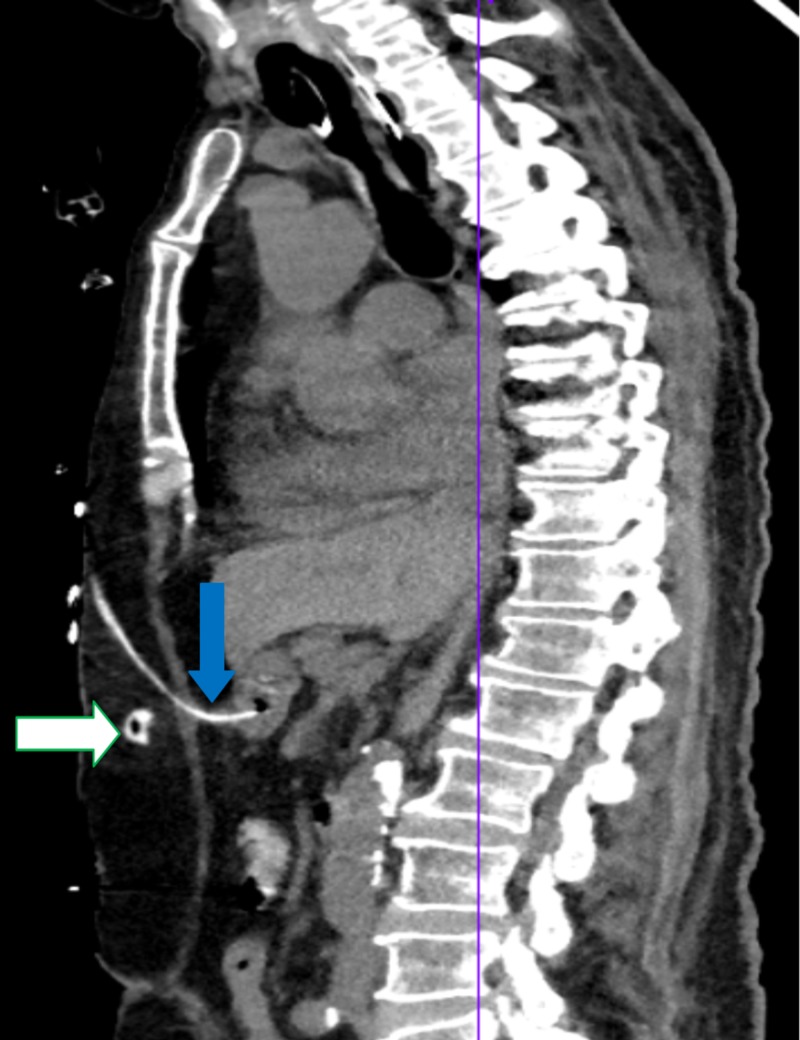
CT of the abdomen/pelvis: VP shunt terminating inside gastric lumen (blue arrow) alongside displaced PEG tube (white arrow) CT, computed tomography; VP, ventriculoperitoneal; PEG, percutaneous endoscopic gastrostomy

**Figure 3:**
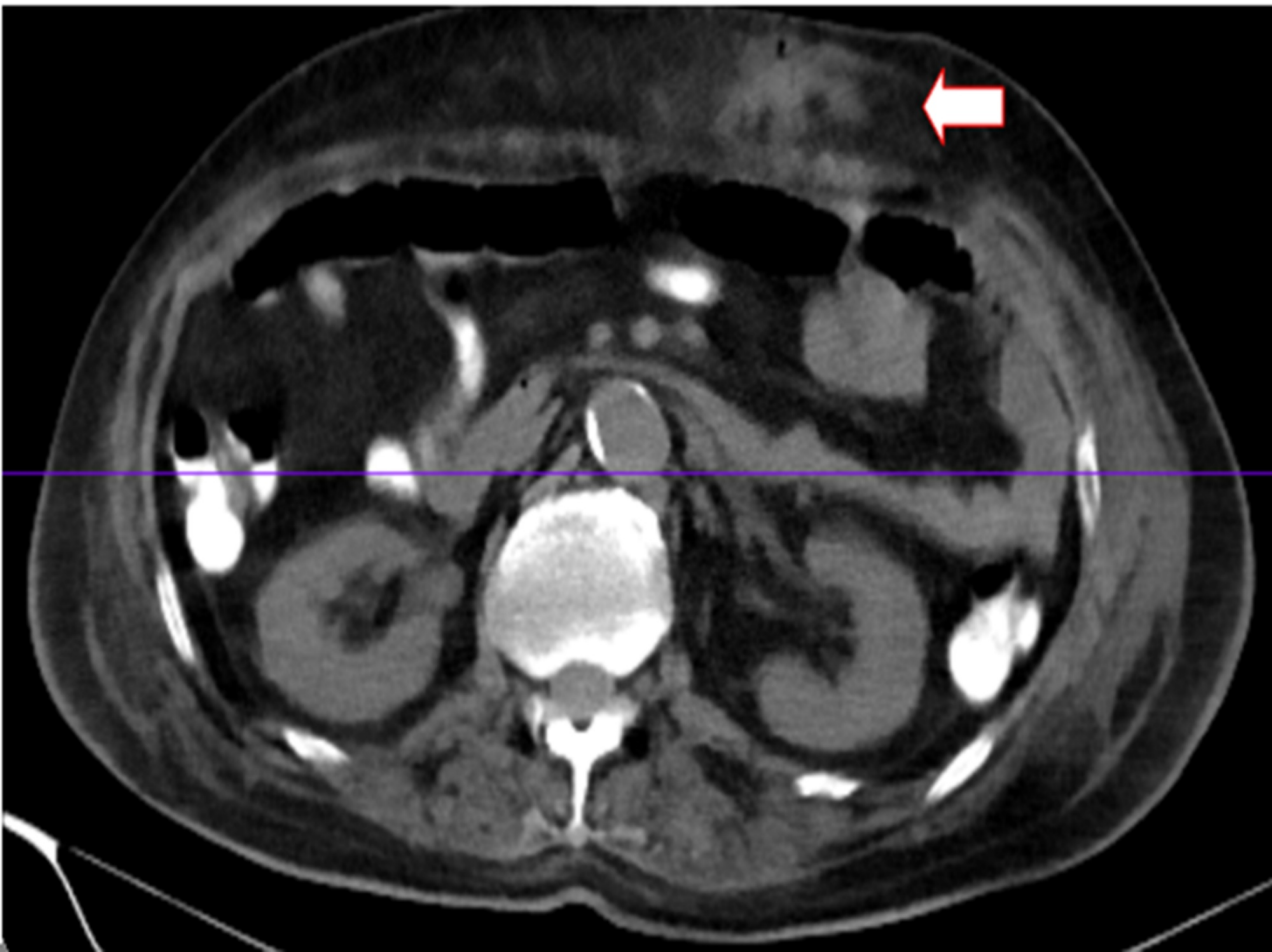
CT abdomen/pelvis: abdominal wall cellulitis with an abscess at PEG insertion site CT, computed tomography; PEG, percutaneous endoscopic gastrostomy

A non-contrast computed tomography (CT) of the chest, abdomen, and pelvis revealed bibasilar pulmonary atelectasis without focal infiltrate and the presence of a right-sided VP shunt catheter traversing the right neck, the right chest, and the right abdominal wall; the tip of the catheter was located within the gastric lumen and had likely entered through the PEG tube insertion site (Figures 1-2). The PEG tube was outside the gastric lumen, terminating in the abdominal wall that was evidenced in the repeat CT scan confirmed that patient had abdominal wall cellulitis and localized abscesses around the PEG tube insertion site (Figure 3). Medical records from another facility confirmed previously normal positioning of the PEG tube and normal intraperitoneal positioning of VP shunt catheter one year prior.

Given the malposition of the VP shunt inside the gastric lumen, we suspected VP shunt infection or meningitis/encephalitis and subsequently lumbar puncture was performed; the results of the cerebrospinal fluid (CSF) analysis were unremarkable. Blood cultures and urine culture results were negative.

The wound culture was positive for Proteus mirabilis sensitive to carbapenems and piperacillin/tazobactam. We debrided the abdominal wall and drained the abscess. Intravenous antibiotic coverage was continued according to the sensitivity testing, and patient received intravenous meropenem for a total of 10 days with good clinical outcome.

**Figure 4:**
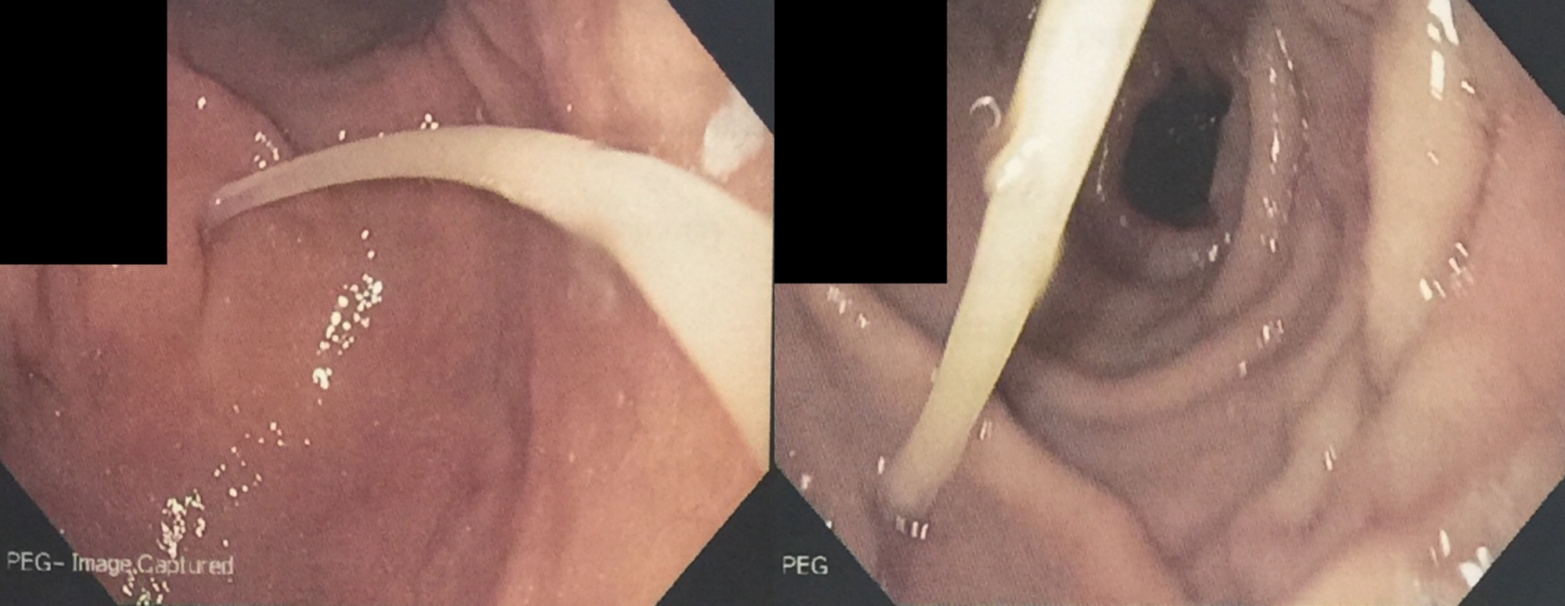
PEG tube insertion site with catheter emerging inside the gastric lumen PEG, percutaneous endoscopic gastrostomy

**Figure 5:**
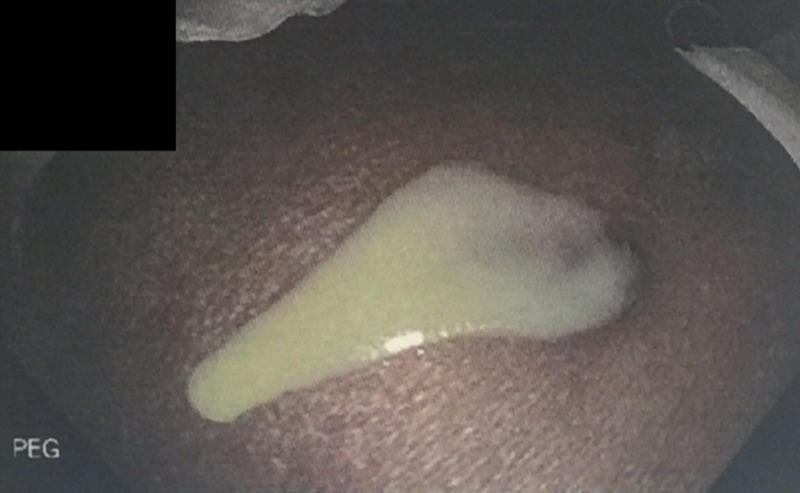
Purulent discharge from PEG tube insertion site PEG, percutaneous endoscopic gastrostomy

Esophagogastroduodenoscopy revealed a small lumen catheter, likely the VP shunt, entering into the gastric lumen proximal to the incisura angularis; this was identified as the previous PEG tube insertion site (Figure 4). The catheter tip was found in the fundus of the stomach. We also noted frank purulent drainage from the PEG insertion site (Figure 5).

Once stabilized, the patient was transferred to a higher center of care for neurosurgical evaluation with proper positioning and removal of the VP shunt. The patient underwent shunt catheter removal from the stomach lumen followed by repositioning of the PEG tube. The neurosurgery team decided against reinsertion of the VP shunt.

The patient was followed up at six months from the time of discharge, and his PEG tube was functional and also he didn't develop worsening of hydrocephalus.

## Discussion

Several complications are associated with VP shunt placement, common among them are shunt obstruction and shunt infections, shunt over drainage/under drainage, and intra-abdominal complications such as abdominal pseudocyst formation and bowel perforation [1]. Usually, shunt placement in adults is associated with a relatively lower risk of complications compared to shunt placement in children [7-8]. Shunt migration has been reported to include scrotal migration, colonic perforation, shunt abandoned in the pelvis, fistulization to the umbilicus, intracardiac migration and knotting, peroral extrusion, gastric perforation, bladder perforation, CSF leakage in the neck, pulmonary vasculature migration, breast migration with CSF galactorrhea, intestinal perforation, pneumonia caused by the transdiaphragmatic erosion, and liver perforation [9]. The reported frequency of bowel perforation is 0.1% to 0.7%, and the colon is the most common site of perforation [1-3]. Regardless of its low incidence rate, the mortality rate due to complications from V-P shunt migration is approximately 15% [2].

To our knowledge, there are only 10 published case reports of gastric perforation by the VP shunt [4-6]. The proposed pathophysiology is that the interaction of the catheter tip with the bowel wall leads to local inflammatory changes and fibrosis causing catheter adhesion to the bowel and delayed perforation with gradual advancement of the catheter inside the lumen [6]. Silicone allergy leading to foreign body reaction may be an alternate mechanism for bowel wall penetration as is infection and manipulation of the bowel during surgery [10]. Unlike previous cases, the likely mechanism of VP shunt migration in our patient was dislodgment of the older PEG tube from the stomach into the abdominal wall, allowing VP shunt migration through intragastric PEG tube tract [4].

The usual presentation of intragastric migration is abdominal pain and gastrointestinal bleeding associated with stomach wall perforation [4-5]. A high degree of clinical suspicion is warranted for diagnosis as only approximately 25% of the patients present with the signs of peritonitis [11]. Intracranial infections such as meningitis caused by enteric organisms like *E. coli* in patients who have undergone previous VP shunt placement should also be investigated for possible shunt migration [12]. The diagnosis is made via radiography (e.g., CT) of the abdomen and pelvis. Upper gastrointestinal endoscopy could be done to determine the specific site of perforation for surgical management [2-3].

Catheter migration is managed both conservatively and surgically. In cases of uncomplicated migration with no peritoneal signs or evidence of CSF infection, the catheter could be left alone and observed as the presence of fibrous sheath around the catheter could prevent leakage of CSF or retrograde flow of gastric contents into the shunt catheter [2,6]. Surgical options for distal catheter management include endoscopic clipping of the distal catheter, direct repair of the stomach wall by laparotomy, and injection of fibrin glue into the catheter tract to repair the perforation [2,4,6]. In our case, the displaced PEG tube was removed followed by neurosurgical removal of the shunt catheter. Appropriate coverage with antibiotics is essential because shunt migration associated with bowel perforation has a very high chance of catheter infection and meningitis with subsequent high mortality. 

## Conclusions

Intragastric migration of a VP shunt is a very rare complication and can be spontaneous or iatrogenic. As in our case, PEG tube placement in these patients may be associated with shunt migration, and the risk of catheter migration needs to be taken into consideration while placing PEG tubes.

## References

[1] Paff M, Alexandru-Abrams D, Muhonen M, Loudon W (2018). Ventriculoperitoneal shunt complications: a review. Interdiscip Neurosurg.

[2] Sathyanarayana S, Wylen E, Baskaya M, Nanda A (2000). Spontaneous bowel perforation after ventriculoperitoneal shunt surgery: case report and a review of 45 cases. Surg Neurol.

[3] Yousfi MM, Jackson NS, Abbas M, Zimmerman RS, Fleischer DE (2003). Bowel perforation complicating ventriculoperitoneal shunt: case report and review. Gastrointest Endosc.

[4] Masuoka J, Mineta T, Kohata T, Tabuchi K (2005). Peritoneal shunt tube migration into the stomach—case report. Neurol Med Chir.

[5] Cheng JY, Lo WC, Liang HH, Kun IH (2007). Migration of ventriculoperitoneal shunt into the stomach, presenting with gastric bleeding. Acta Neurochir.

[6] Cohen-Addad DI, Hewitt K, Bell D (2018). A ventriculoperitoneal shunt incidentally found in the stomach. Radiol Case Rep.

[7] Wu Y, Green NL, Wrensch MR, Zhao S, Gupta N (2007). Ventricluoperitoneal shunt complications in California: 1990 to 2000. Neurosurgery.

[8] Reddy GK, Bollam P, Caldito G (2014). Long term outcomes of ventriculoperitoneal shunt surgery in patients with hydrocephalus. World Neurosurg.

[9] Ricci C, Velimirovic BM, Fitzgerald TN (2016). Case report of migration of 2 ventriculoperitoneal shunt catheters to the scrotum: Use of an inguinal incision for retrieval, diagnostic laparoscopy and hernia repair. Int J Surg Case Rep.

[10] Brownlee JD, Brodkey JS, Schaefer IK (1998). Colonic perforation by ventriculoperitoneal shunt tubing: A case of suspected silicone allergy. Surg Neurol.

[11] Sells Sells, CJ CJ, Looser JD (1973). Peritonitis following perforation of the bowel: A rare complication of Ventriculoperitoneal shunt. J Pediatr.

[12] Abu-Dalu K, Pode D, Hadani M, Safar A (1983). Colonic complications of ventriculoperitoneal shunts. Neurosurg.

